# Factors associated with follow-up care after pediatric concussion: A longitudinal population-based study in Alberta, Canada

**DOI:** 10.3389/fped.2022.1035909

**Published:** 2023-01-09

**Authors:** Krystle Wittevrongel, Olesya Barrett, Brent E. Hagel, Kathryn J. Schneider, David W. Johnson, Keith Owen Yeates, Jennifer D. Zwicker

**Affiliations:** ^1^ School of Public Policy, University of Calgary, Calgary, AB, Canada; ^2^ Alberta Health Services, Calgary, AB, Canada; ^3^ O’Brien Institute for Public Health, University of Calgary, Calgary, AB, Canada; ^4^ Department of Pediatrics, Cumming School of Medicine, University of Calgary, Calgary, AB, Canada; ^5^ Department of Community Health Sciences, Cumming School of Medicine, University of Calgary, Calgary, AB, Canada; ^6^ Alberta Children’s Hospital Research Institute, University of Calgary, Calgary, AB, Canada; ^7^ Sport Injury Prevention Research Centre, Faculty of Kinesiology, University of Calgary, Calgary, AB, Canada; ^8^ Faculty of Kinesiology, University of Calgary, Calgary, AB, Canada; ^9^ Hotchkiss Brain Institute, University of Calgary, Calgary, AB, Canada; ^10^ Department of Psychology, University of Calgary, Calgary, AB, Canada

**Keywords:** pediatric, concussion, children, youth, MTBI, longitudinal, epidemiology, follow-up

## Abstract

**Background:**

Concussion is a common injury in children and adolescents. Current best practice guidelines indicate that recovery should be supervised through recurrent follow-up visits. A more detailed understanding of the system-level and individual factors that are associated with follow-up care is a critical step towards increasing evidence-based practice. The objective of this study was to identify predisposing, enabling, and need-based factors associated with follow-up care after pediatric concussion.

**Materials and methods:**

A retrospective population-based cohort study was conducted using linked, province-wide administrative health data for all patients <18 years of age with a diagnosis of concussion, other specified injuries of the head, unspecified injury of head, or post-concussion syndrome (PCS) between April 1, 2004 and March 31, 2018 in Alberta, Canada. The association between predisposing, enabling, and need-based factors and the receipt of follow-up care within a defined episode of care (EOC) was analyzed using logistic regression models for the entire cohort and for EOC that began with a concussion diagnosis. Predisposing factors included age and sex. Enabling factors included the community type of patient residence, area-based socioeconomic status (SES), and visit year. Need-based factors included where the EOC began (outpatient vs. emergency settings) and history of previous concussion-related EOC.

**Results:**

194,081 EOCs occurred during the study period but only 13% involved follow-up care (*n* = 25,461). Males and adolescents were more likely to receive follow-up care. Follow-up was less likely among patients who lived in remote communities or in areas of lower SES, while EOCs beginning in 2011 or later were more likely to involve follow-up care. Patients whose EOC began in outpatient settings, had more than one EOC, or a diagnosis of concussion were more likely to receive follow-up care.

**Conclusion:**

Follow-up care for pediatric concussion has increased over time and is associated with patient age and sex, history of concussion-related EOC, where a patient lives (community type and area-based SES), and when and where the index visit occurs. A better understanding of which children are more likely to receive follow-up care, as well as how and when they do, is an important step in aligning practice with follow-up guidelines.

## 1. Introduction

Concussion is a common injury in children and adolescents as indicated by high rates of health care use (1). Post-concussive sequelae have the potential to persist for months to years following injury, resulting in a myriad of negative physical, emotional, cognitive, and behavioural outcomes (2), which can be especially prolonged if the concussion is unmanaged (3–5). Current best practice guidelines indicate that patient recovery should be supervised through recurrent physician follow-up visits (6). Substantial practice variation and knowledge gaps in concussion management have been reported by pediatric emergency department (ED) physicians and community physicians (7, 8). Although a lack of follow-up care consistent with recommended guidelines has been reported, direct evidence on follow-up visits is limited (9–11). Our recent study indicated a trend towards increasing rates of follow-up care over time but also showed that the overall rate of follow-up visits fell below levels expected if practice was aligned with guidelines (1). A more detailed understanding of the system-level and individual factors that are associated with follow-up care is a critical step towards increasing evidence-based practice (12, 13).

This study focused on identifying factors that facilitate or impede follow-up care for pediatric concussion. The Andersen Behavioural Model of Health Service Use guided analyses of individual and system-level factors that influence health service utilization of follow-up care after pediatric concussion (13–15). This model of health service use identifies factors in three categories: those that predispose an individual or population towards service use, those that enable service use, and the individual or population's need for care (14, 15). Predisposing factors reflect the propensity of certain individuals to use services. Enabling factors are those that facilitate health service use and make health resources available (14). Factors related to need include both perceived need (subjective need or care seeking and adherence to guidelines or recommendations) and evaluated need (objective need or the type and amount of treatment that will be provided after a patient has presented for care) related to health service use (15).

To identify individual and system-level factors that facilitated or impeded follow-up care for pediatric concussion, we used province-wide administrative health data to characterize health care utilization in episodes of care (EOC) that included vs. did not include follow-up care among children and youth following a concussion-related diagnosis in Alberta, Canada from 2004 to 2018. Due in part to universal taxpayer-funded physician and hospital health care services and the linkage of databases capturing all inpatient and outpatient services, Alberta's health administrative databases serve as a valuable population-based repository of all pediatric concussion-related diagnoses across the province. The objective of this study was to identify predisposing, enabling, and need-based factors associated with follow-up care after pediatric concussion.

## 2. Methods

This study received ethics approval from the University of Calgary (REB17-1957_REN3), administrative approval from Alberta Health Services (AHS), and is reported according to STROBE guidelines (16). A retrospective population-based cohort design was employed using linked administrative health data. Data sources included the Alberta Ambulatory Care Reporting System (AACRS), National Ambulatory Care Reporting System (NACRS), and physician claims databases. These databases were deterministically linked using unique encoded identifiers based on patient Personal Health Number/Unique Lifetime Identifier (PHN/ULI) to identify pediatric patients (anyone <18 years of age) who received publicly-funded care for concussion-related injuries between April 1, 2004 and March 31, 2018 in ED or physician office (PO) settings in Alberta, Canada (1).

### 2.1. Definitions and study variables

We utilized the Centers for Disease Control and Prevention administrative data definition in place when the study commenced to identify concussion codes for inclusion (17). This definition allows for the identification of concussion for surveillance purposes when reviewing coded health care administrative databases (17). The exact case definition is detailed elsewhere (1). In brief, International Statistical Classification of Diseases and Related Health Problems, 9th Revision (ICD-9) and 10th Revision (ICD-10) diagnostic codes for concussion, post-concussion syndrome (PCS), other specified injuries of head, and unspecified injury of head were included (Table 1).

**Table 1:** Predisposing factors, enabling factors, and need-based factors used as explanatory variables as guided by the Andersen Behavioural Model of Health Service Use (13–15).

Factor Category	Definition
**Predisposing Factors**	
Age	Patient age (actual; years) at time of index visit
Sex	Patient sex (male/female) at time of index visit
**Enabling Factors**	
Community Type of Patient Residence	Patient residence was defined along the urban-rural continuum by community size, denoted by one of seven categories developed by AHS: metro centres (populations over 500,000 – i.e., Calgary and Edmonton), metro-influenced areas (areas immediately surrounding Calgary and Edmonton and deemed commuter communities), urban areas (population between 25,000 and 500,000 – five in total), urban-influenced areas [Local Geographic Areas (LGA) surrounding the five urban areas], large rural centres and surrounding areas (populations between 10,000 and 25,000), rural areas (populations less than 10,000 and up to 200 km from a metro or urban centre), and remote areas (greater than 200 km from a metro or urban centre) (19).
Patient Area-Based Socioeconomic Status (SES)	The Pampalon Deprivation Index (PDI) is the most widely used and cited area-based socioeconomic indicator in Canada (19) and was used to denote SES in this study. The PDI stratifies the population into five quintiles depending on the level of deprivation, from the lowest level of deprivation (PDI = 1, most advantaged) to the highest level of deprivation (PDI = 5, least advantaged).
Visit Year	We defined visit year based on the data of the index visit of the EOC. That date was coded depending on whether it occurred between 2004–2010 or 2011–2018 to align with previous research that indicates increased visit rates for concussion were observed over time, particularly after 2010 (9, 35).
**Factors Related to Need**	
Diagnosis	We used the Centers for Disease Control and Prevention administrative data definition for research in place when the study commenced to define concussion codes (17). ED visits were captured in AACRS and NACRS and occurred in advanced ambulatory care centres, ED settings, and urgent care centres. These visits were defined as an International Statistical Classification of Diseases and Related Health Problems, 10th Revision (ICD-10) diagnosis of F07.2 (“postconcussional syndrome”), S09.8 (“other specified injuries of head”), S09.9 (“unspecified injury of head”), or one of 13 concussion diagnostic codes (S06.0, S06.000, S06.001, S06.010, S06.011, S06.020, S06.021, S06.030, S06.031, S06.040, S06.041, S06.090, S06.091). Visits captured in physician claims data took place in outpatient settings, including primary care physicians, medical specialists, and other non-emergency clinics and were defined as an International Statistical Classification of Diseases and Related Health Problems, 9th Revision (ICD-9) diagnosis of 850 (“concussion”) or 310.2 (“postconcussional syndrome”).
Location of Health Service Use: Emergency Department (ED) or Physician Office (PO)	Visits to advanced ambulatory care centres, emergency departments, and urgent care centres were defined as ED visits, while visits to outpatient settings, including physician offices and other non-urgent clinics, were defined as PO visits. A maximum of ten ICD-10 diagnostic codes per ED visit were recorded and submitted by professional coders. A maximum of three ICD-9 codes per PO visit were recorded and submitted by physicians or their delegates.
History of Concussion-Related EOC	Patients with visits that could be linked to additional EOC were considered as having multiple EOC. Cases where the maximum number of EOC is one were considered a single EOC.

An EOC definition was used to differentiate follow-up visits from new injuries in children and youth (children hereafter) with multiple concussion-related health care visits. Our definition was developed in conjunction with study clinicians and informed by previous population-based studies with similar approaches (10, 18). An index visit to ED or PO was defined as the start of an EOC. An EOC is considered a new injury if, for an ED visit, more than 30 days had passed since a previous visit (to ED or PO), and, for a PO visit, more than 90 days had passed since a previous visit (to ED or PO). If a concussion-related visit occurred within these timelines, it was designated a follow-up visit and assigned to the preceding EOC. Preliminary descriptive analyses indicated that the time between initial and follow-up visits was clustered around these time points.

Often, variables of interest cannot be abstracted from administrative health data. As such, the Andersen Behavioural Model of Health Service Use guided the selection of factors that may be associated with follow-up care in children and youth after a concussion. Predisposing factors included patient age and sex. Enabling factors consisted of the community type of the patient's residence as defined by Alberta Health Services (AHS) (19), the area-based socioeconomic status associated with the patient's residence as defined by the Pampalon Deprivation Index (PDI) (20), and the visit year (Table 1). Need-based factors included diagnosis, location of treatment (ED or PO), and whether the patient had a history of any previous concussion-related EOC (Table 1). There was no consistent indicator of severity coded in both ED and PO and injury severity scores were not derived.

### 2.2. Statistical analysis

Descriptive measures were used to summarize all predisposing, enabling, and need-based factors in EOC that included vs. did not include follow-up care. Those EOC that began with a diagnosis of concussion were also examined separately in a sub-analysis. The association between the factors identified (Table 1) and follow-up care was then analyzed using logistic regression models: first univariate then multivariable analyses, adjusting for statistically significant factors. Adjustment was made for the clustering of individuals to account for the fact that an individual may have had more than a single EOC. The location of health service use was included as a predictor only in the concussion sub-analysis as only concussion and post-concussion syndrome diagnoses were coded in both ED and PO.

Multicollinearity was assessed using the variance inflation factor (VIF), model discrimination was evaluated *via* receiver operating characteristic (ROC) curves, the final model was calibrated by testing for specification error, and the Hosmer-Lemeshow test was used to assess goodness of fit. The linearity of log odds was evaluated for continuous variables. An *α* = 0.05 level was set *a priori* as the threshold for significance. Data were analyzed using Stata 15.1 (21).

## 3. Results

Details regarding cohort creation are reported elsewhere (1). In brief, the study cohort consisted of 162,982 unique children (<18 years of age) who received medical care for the included diagnoses in a total of 230,575 visits constituting 194,081 EOC. The cohort was primarily male and from metropolitan, metro-influenced, or rural areas (Table 2). Nearly half of all EOC occurred in children under 10 years of age (Table 2). Just over half of all EOC involved a diagnosis of concussion (Table 2), and the median age at the start of a concussion EOC was older than that for other diagnoses (Table 3). Males were more common among children with a concussion diagnosis, as were children residing in metropolitan, metro-influenced, and rural areas, relative to children with a non-concussion diagnosis (Table 2). Children living in areas of mid to high socioeconomic status (SES; PDI of 2 or 3) accounted for more EOC overall, especially for those with a concussion diagnosis (Table 2). Concussion diagnoses occurred more often in ED and among patients with at least one other EOC when compared with children with other non-concussion diagnoses (Table 2). The cohort had a mean of 1.5 visits per EOC and 1.2 EOC per patient. These rates were higher in PO and for children with a concussion diagnosis (Table 3).

**Table 2:** Patient demographics for pediatric concussion, post-concussion syndrome, other specified injuries of head, and unspecified injury of head EOC occurring in ED or PO settings between April 1, 2004 and March 31, 2018 in Alberta.

	Total Cohort	Concussion Diagnosis	Other (Non-Concussion) Diagnoses	Post-Concussion Syndrome Diagnosis
	*N* = 194,081 (%)	*n* = 101,677 (%)	*n* = 87,212 (%)	*n* = 5,192 (%)
**Predisposing Factors**				
*Sex*				
Male	118,065 (60.8%)	64,054 (63.0%)	52,677 (58.6%)	2,945 (56.7%)
Female	76,016 (39.2%)	37,623 (37.0%)	37,242 (41.4%)	2,247 (43.3%)
*Age at Index Visit (years)*				
<1	13,839 (7.1%)	2,049 (2.0%)	11,776 (13.5%)	14 (0.3%)
1	16,714 (8.6%)	3,537 (3.5%)	13,142 (15.1%)	35 (0.7%)
2	11,487 (5.9%)	3,026 (3.0%)	8,436 (9.7%)	25 (0.5%)
3	8,556 (4.4%)	2,560 (2.5%)	5,948 (6.8%)	48 (0.9%)
4	6,568 (3.4%)	2,223 (2.2%)	4,303 (4.9%)	42 (0.8%)
5	6,430 (3.3%)	2,592 (2.5%)	3,768 (4.3%)	70 (1.4%)
6	6,616 (3.4%)	3,031 (3.0%)	3,500 (4.0%)	85 (1.6%)
7	6,609 (3.4%)	3,314 (3.3%)	3,181 (3.6%)	114 (2.2%)
8	6,848 (3.5%)	3,743 (3.7%)	2,949 (3.4%)	156 (3.0%)
9	7,506 (3.9%)	4,469 (4.4%)	2,879(3.3%)	158 (3.0%)
10	8,434 (4.3%)	5,219 (5.1%)	2,980 (3.4%)	235 (4.5%)
11	9,734 (5.0%)	6,410 (6.3%)	3,056 (3.5%)	268 (5.2%)
12	10,713 (5.5%)	7,256 (7.1%)	3,142 (3.6%)	315 (6.1%)
13	13,019 (6.7%)	9,093 (8.9%)	3,423 (3.9%)	503 (9.7%)
14	14,435 (7.4%)	10,280 (10.1%)	3,522 (4.0%)	633 (12.2%)
15	15,924 (8.2%)	11,424 (11.2%)	3,733 (4.3%)	767 (14.8%)
16	16,016 (8.3%)	11,330 (11.1%)	3,797 (4.4%)	889 (17.1%)
17	14,633 (7.5%)	10,121 (10.0%)	3,677 (4.2%)	835 (16.1%)
**Enabling Factors**				
*Community Type (Patient Residence)*				
Metropolitan	82,550 (42.5%)	44,279 (43.6%)	35,848 (41.1%)	2,423 (46.7%)
Metro-Influenced Areas	33,432 (17.2%)	19,405 (19.1%)	13,007 (14.9%)	1,020 (19.7%)
Urban	18,782 (9.7%)	8,727 (8.6%)	9,699 (11.1%)	356 (6.9%)
Urban-Influenced Areas	5,082 (2.6%)	2,876 (2.8%)	2,075 (2.4%)	131 (2.5%)
Rural	38,534 (19.9%)	19,654 (19.3%)	17,943 (20.6%)	937 (18.1%)
Large Rural Centres and Surrounding Areas	9,561 (4.9%)	4,626 (4.6%)	4,678 (5.4%)	257 (5.0%)
Remote	6,140 (3.2%)	2,110 (2.1%)	3,962 (4.5%)	68 (1.3%)
*Patient Socioeconomic Status (SES)* ^a^				
PDI = 1	33,361 (17.3%)	18,397 (18.1%)	14,094 (16.8%)	1,140 (22.7%)
PDI = 2	41,202 (21.2%)	22,635 (22.3%)	17,393 (20.8%)	1,174 (23.4%)
PDI = 3	39,054 (20.1%)	20,362 (20.0%)	17,699 (21.2%)	993 (19.8%)
PDI = 4	36,692 (18.9%)	19,208 (18.9%)	16,555 (19.8%)	929 (18.5%)
PDI = 5	36,366 (18.7%)	17,627 (17.3%)	17,956 (21.4%)	783 (15.6%)
*Visit Year*				
2004–2010	70,079 (36.1%)	33,831 (33.3%)	35,049 (40.2%)	1,199 (23.1%)
2011–2018	124,002 (63.9%)	67,846 (66.7%)	52,163 (59.8%)	3,993 (76.9%)
**Factors Related to Need**				
*History of Concussion-Related EOC*				
Single EOC	138,542 (71.4%)	68,937 (67.8%)	66,749 (76.5%)	2,856 (55.0%)
>1 EOC	55,539 (28.6%)	32,740 (32.2%)	20,463 (23.5%)	2,336 (45.0%)
*Location of Index Visit*				
ED	n/a	61,465 (60.5%)	87,212 (100.0%)	1,879 (36.2%)
PO	n/a	40,212 (39.5%)	n/a	3,313 (63.8%)
*Diagnosis*				
Concussion	101,677 (52.4%)	101,677 (100.0%)	n/a	n/a
Other specified injuries of head	8,547 (4.4%)	n/a	8,547 (9.8%)	n/a
Postconcussional syndrome	5,192 (2.7%)	n/a	n/a	5,192 (100.0%)
Unspecified injury of head	78,665 (40.5%)	n/a	78,665 (90.2%)	n/a

^a^ Does not sum to 100.0% due to the exclusion of small dissemination areas in the calculation of the PDI.

**Table 3:** Descriptive statistics on EOC and follow-up visits for cohort and by diagnosis for pediatric concussion-related visits to ED or PO settings in Alberta between April 1, 2004 and March 31, 2018.

	Overall					ED					PO				
	**Min**	**Max**	**Mean**	**Median**	**Standard Deviation**	**Min**	**Max**	**Mean**	**Median**	**Standard Deviation**	**Min**	**Max**	**Mean**	**Median**	**Standard Deviation**
Cohort															
Total number of visits per EOC	1	18	1.5	1	1.2	1	18	1.2	1	0.7	1	18	2.1	2	1.7
Total number of EOC per unique patient	1	10	1.2	1	0.6	1	10	1.2	1	0.5	1	9	1.3	1	0.7
Number of days since previous EOC	31	5,022	916.5	612	896.3	31	5,022	934.2	645	896.7	91	4,950	873.8	534	893.9
Number of days last concussion-related visit in EOC	0	90	10.9	6	14.7	1	30	3.4	1	5.8	0	90	14.1	8	16
Age at start of EOC	0	17	9.1	10	5.8	0	17	8.5	9	5.8	0	17	11.3	13	4.9
Age at follow-up	0	17	12.8	14	4.2	0	17	10.7	12	5.3	0	17	13.6	14	3.3
Concussion Diagnosis															
Total number of visits per EOC	1	18	1.7	1	1.3	1	18	1.4	1	0.8	1	18	2	2	1.6
Total number of EOC per unique patient	1	9	1.3	1	0.6	1	7	1.2	1	0.6	1	9	1.3	1	0.7
Number of days since previous EOC	31	5,022	953.0	640	921.3	31	5,022	987.4	691	928	91	4,950	902.7	565	909.1
Number of days last concussion-related visit in EOC	0	90	11.1	7	14.7	0	30	2.9	1	5.2	0	90	13.5	8	15.7
Age at start of EOC	0	17	11.3	13	4.8	0	17	11.4	13	4.7	0	17	11.2	13	5
Age at follow-up	0	17	13.1	14	3.8	0	17	11.6	13	4.7	0	17	13.5	14	3.3
Other (Non-Concussion) Diagnoses^a^ ED Only															
Total number of visits per EOC	1	15	1.1	1	0.4	1	15	1.1	1	0.4	n/a
Total number of EOC per unique patient	1	10	1.1	1	0.4	1	10	1.1	1	0.4
Number of days since previous EOC	31	5,012	887.8	605	855.9	31	5,012	887.8	605	855.9
Number of days last concussion-related visit in EOC	0	30	3.2	1	6.1	0	30	3.2	1	6.1
Age at start of EOC	0	17	6.3	5	5.6	0	17	6.3	5	5.6
Age at follow-up	0	17	7.7	7	5.9	0	17	7.7	7	5.9
Post-Concussion Syndrome Diagnosis															
Total number of visits per EOC	1	16	2.5	2	2	1	15	2.1	2	1.5	1	16	2.7	2	2.2
Total number of EOC per unique patient	1	8	1.5	1	0.8	1	7	11.4	1	0.7	1	8	1.5	1	0.8
Number of days since previous EOC	31	4,831	663.7	345	778.6	31	4,659	639.8	300	830.9	91	4,831	675.1	365	752.3
Number of days last concussion-related visit in EOC	0	90	14.6	9	16.2	0	30	6.9	4	7.1	0	90	17.1	11	17.5
Age at start of EOC	0	17	13.3	14	3.6	0	17	13	14	3.7	0	17	13.5	14	3.5
Age at follow-up	0	17	13.6	14	3.3	0	17	13.1	14	3.5	0	17	13.8	15	3.2

^a^ Non-concussion diagnoses include other specified injuries of head und unspecified injury of head.

### 3.1. Follow-up care

Of the 194,081 total EOC, 13% involved follow-up care (*n* = 25,461), encompassing 36,494 visits (some EOC involved more than one follow-up visit) involving 23,499 children (some children had more than one EOC) (Tables 4, 5). Follow-up care became more common over time, and the proportion of EOC with at least one follow-up visit increased from 9% to 16% over the study period. For concussion EOC, the proportion increased from 16% to 22%, whereas for other non-concussion head injuries, the increase was from 4% to 6%. The median age of follow-up was 14 years and two-thirds of all follow-up visits involved adolescents aged 13–17 years (Tables 3, 4). Follow-up care was more frequent in males (62%), in children living in metropolitan or metro-influenced areas (64%), and among those living in areas of mid-high SES (Table 4). Follow-up visits were concentrated in PO settings and in the latter half of the study period (Table 4). The mean interval between visits in an EOC was 10.9 days, although this was longer in PO settings and for children with a concussion diagnosis (Table 3). Over three-quarters of all follow-up visits involved a diagnosis of concussion (Table 4). Table 5 shows the demographic differences between EOC with vs. without follow-up care.

**Table 4:** Patient demographics for pediatric concussion, post-concussion syndrome, other specified injuries of head, and unspecified injury of head follow-up visits occurring in ED or PO settings between April 1, 2004 and March 31, 2018 in Alberta.

	Total Cohort	Concussion Diagnosis	Other (Non-Concussion) Diagnoses	Post-Concussion Syndrome Diagnosis
	*n* = 36,494 (%)	*n* = 28,316 (%)	*n* = 2,955 (%)	*n* = 5,223 (%)
*Total number of follow-up visits in EOC*				
1	25,461 (69.8%)	19,729 (69.7%)	2,717 (91.9%)	3,015 (57.7%)
2	6,526 (17.9%)	5,166 (18.2%)	199 (6.7%)	1,161 (22.2%)
3	2,284 (6.3%)	1,743 (6.2%)	26 (0.9%)	515 (9.9%)
4	991 (2.7%)	759 (2.7%)	3 (0.1%)	229 (4.4%)
>5	1,232 (3.4%)	919 (3.2%)	10 (0.3%)	303 (5.8%)
*Sex*				
Male	22,515 (61.7%)	17,869 (63.1%)	1,755 (59.4%)	2,891 (55.4%)
Female	13,979 (38.3%)	10,447 (36.9%)	1,200 (40.6%)	2,332 (44.6%)
*Age (years)*				
<1	495 (1.4%)	197 (0.7%)	290 (9.8%)	8 (0.2%)
1	748 (2.1%)	368 (1.3%)	369 (12.5%)	11 (0.2%)
2	560 (1.5%)	309 (1.1%)	243 (8.2%)	8 (0.2%)
3	476 (1.3%)	295 (1/0%)	165 (5.6%)	16 (0.3%)
4	393 (1.1%)	254 (0.9%)	116 (3.9%)	23 (0.4%)
5	427 (1.2%)	282 (1.0%)	111 (3.8%)	34 (0.7%)
6	539 (1.5%)	374 (1.3%)	109 (3.7%)	56 (1.1%)
7	718 (2.0%)	522 (1.8%)	110 (3.7%)	89 (1.7%)
8	933 (2.6%)	708 (2.5%)	97 (3.3%)	128 (2.5%)
9	1,053 (2.9%)	827 (2.9%)	102 (3.5%)	124 (2.4%)
10	1,476 (4.0%)	1,173 (4.1%)	116 (3.9%)	187 (3.6%)
11	2,040 (5.6%)	1,623 (5.7%)	122 (4.1%)	295 (5.7%)
12	2,607 (7.1%)	2,112 (7.5%)	133 (4.5%)	362 (6.9%)
13	3,449 (9.5%)	2,802 (9.9%)	152 (5.1%)	495 (9.5%)
14	4,469 (12.3%)	3,603 (12.7%)	171 (5.8%)	695 (13.3%)
15	5,508 (15.1%)	4,479 (15.8%)	192 (6.5%)	837 (16.0%)
16	5,507 (15.1%)	4,417 (15.6%)	194 (6.6%)	896 (17.2%)
17	5,096 (14.0%)	3,971 (14.0%)	163 (5.5%)	962 (18.4%)
*Community Type (Patient Residence)*				
Metropolitan	15,097 (41.4%)	11,686 (41.3%)	1,082 (36.6%)	2,329 (44.6%)
Metro-Influenced Areas	8,308 (22.8%)	6,595 (23.3%)	667 (22.6%)	1,046 (20.0%)
Urban	2,785 (7.6%)	2,232 (7.9%)	218 (7.4%)	335 (6.4%)
Urban-Influenced Areas	1,007 (2.8%)	803 (2.8%)	66 (2.2%)	138 (2.6%)
Rural	6,855 (18.8%)	5,128 (18.1%)	667 (22.6%)	1,060 (20.3%)
Large Rural Centres and Surrounding Areas	1,703 (4.7%)	1,341 (4.7%)	133 (4.5%)	229 (4.4%)
Remote	739 (2.0%)	531 (1.9%)	122 (4.1%)	86 (1.7%)
*Patient Socioeconomic Status (SES)* ^a^				
PDI = 1	6,873 (19.5%)	5,313 (19.4%)	418 (0.1%)	1,142 (22.5%)
PDI = 2	8,714 (24.7%)	6,885 (25.1%)	661 (22.4%)	1,168 (23.0%)
PDI = 3	7,403 (21.0%)	5,768 (21.1%)	594 (20.1%)	1,041 (20.5%)
PDI = 4	6,628 (18.8%)	5,092 (18.6%)	544 (18.4%)	992 (19.5%)
PDI = 5	5,690 (16.1%)	4,347 (15.9%)	608 (20.6%)	735 (14.5%)
*Visit Year*				
2004–2010	9,627 (26.4%)	7,181 (25.4%)	1,428 (48.3%)	1,018 (19.5%)
2011–2018	26,867 (73.6%)	21,135 (74.6%)	1,527 (51.7%)	4,205 (80.5%)
*Location of Visit*				
ED	10,733 (29.4%)	6,508 (23.0%)	2,955 (100.0%)	1,270 (24.3%)
PO	25,761 (70.6%)	21,808 (77.0%)	n/a	3,953 (75.7%)

^a^ Does not sum to 100.0% due to the exclusion of small dissemination areas in the calculation of the PDI.

**Table 5:** Demographic characteristics of pediatric concussion, post-concussion syndrome, other specified injuries of head, and unspecified injury of head EOC with follow-up care vs. EOC without follow-up care between April 1, 2004 and March 31, 2018 in Alberta.

	Total Cohort *N* = 194,081 (%)		Concussion Diagnosis *n* = 101,677 (%)		Other (Non-Concussion) Diagnoses *n* = 87,212 (%)	
	No Follow-Up	Received Follow-Up Care	No Follow-Up	Received Follow-Up Care	No Follow-Up	Received Follow-Up Care
	*n* = 168,620 (86.9%)	*n* = 25,461 (13.1%)	*n* = 81,947 (80.6%)	*n* = 19,730 (19.4%)	*n* = 82,702 (94.8%)	*n* = 4,510 (5.2%)
**Predisposing Factors**						
*Sex*						
Male	102,011 (60.5%)	16,054 (63.1%)	51,426 (62.8%)	12,628 (64.0%)	48,312 (94.6%)	2,754 (5.4%)
Female	66,609 (39.5%)	9,407 (36.9%)	30,521 (37.2%)	7,102 (36.0%)	34,390 (95.1%)	1,756 (4.9%)
*Age at Index Visit (years)*						
Median	9	14	12	14	4	12
<1	13,364 (7.9%)	475 (1.9%)	1,847 (2.3%)	202 (1.0%)	11,505 (13.0%)	271 (6.0%)
1	16,034 (9.5%)	680 (2.7%)	3,207 (3.9%)	330 (1.7%)	12,795 (15.5%)	347 (7.7%)
2	10,980 (6.5%)	507 (2.0%)	2,729 (3.3%)	297 (1.5%)	8,228 (10.0%)	208 (4.6%)
3	8,131 (4.8%)	425 (1.7%)	2,314 (2.8%)	246 (1.3%)	5,773 (7.0%)	175 (3.9%)
4	6,230 (3.7%)	338 (1.3%)	2,025 (2.5%)	198 (1.0%)	4,165 (5.0%)	138 (3.1%)
5	6,055 (3.6%)	375 (1.5%)	2,360 (2.9%)	232 (1.2%)	3,637 (4.4%)	131 (2.9%)
6	6,161 (3.7%)	455 (1.8%)	2,705 (3.3%)	326 (1.7%)	3,383 (4.1%)	117 (2.6%)
7	6,020 (3.6%)	589 (2.3%)	2,877 (3.5%)	437 (2.2%)	3,044 (3.7%)	137 (3.0%)
8	6,148 (3.7%)	700 (2.8%)	3,217 (3.9%)	526 (2.7%)	2,808 (3.4%)	141 (3.1%)
9	6,653 (4.0%)	853 (3.4%)	3,807 (4.7%)	662 (3.4%)	2,715 (3.3%)	164 (3.6%)
10	7,307 (4.3%)	1,127 (4.4%)	4,324 (5.3%)	895 (4.5%)	2,792 (3.4%)	188 (4.2%)
11	8,243 (4.9%)	1,491 (5.9%)	5,193 (6.3%)	1,217 (6.2%)	2,828 (3.4%)	228 (5.1%)
12	8,873 (5.3%)	1,840 (7.2%)	5,761 (7.0.%)	1,495 (7.6%)	2,864 (3.5%)	278 (6.1%)
13	10,587 (6.3%)	2,432 (9.6%)	7,114 (8.7%)	1,979 (10.0%)	3,084 (3.7%)	339 (7.5%)
14	11,445 (6.8%)	2,990 (11.7%)	7,854 (9.6%)	2,426 (12.3%)	3,125 (3.8%)	397 (8.8%)
15	12,346 (7.3%)	3,578 (14.1%)	8,502 (10.4%)	2,922 (14.8%)	3,295 (4.0%)	438 (9.7%)
16	12,447 (7.4%)	3,569 (14.0%)	8,448 (10.3%)	2,882 (14.6%)	3,345 (4.0%)	452 (10.0%)
17	11,596 (6.9%)	3,037 (11.9%)	7,663 (9.4%)	2,458 (12.5%)	3,316 (4.0%)	361 (8.0%)
**Enabling Factors**						
*Community Type (Patient Residence)*						
Metropolitan	72,068 (42.7%)	10,482 (41.2%)	35,968 (43.9%)	8,311 (42.1%)	34,257 (41.2%)	1,591 (35.3%)
Metro-Influenced Areas	27,654 (16.4%)	5,778 (22.7%)	14,829 (18.1%)	4,576 (23.2%)	12,052 (14.6%)	955 (21.2%)
Urban	16,867 (10.0%)	1,915 (7.%)	7,266 (8.9%)	1,461 (7.4%)	9,320 (11.3%)	379 (8.4%)
Urban-Influenced Areas	4,376 (2.6%)	706 (2.6%)	2,312 (2.8%)	564 (2.9%)	1,958 (2.4%)	117 (2.6%)
Rural	33,652 (20.0%)	4,882 (19.%)	16,043 (19.6%)	3,611 (18.3%)	16,892 (20.4%)	1,051 (23.3%)
Large Rural Centres and Surrounding Areas	8,393 (5.0%)	1,168 (4.3%)	3,761 (4.6%)	865 (4.4%)	4,432 (5.4%)	246 (5.4%)
Remote	5,610 (3.3%)	530 (2.0%)	1,768 (2.2%)	342 (1.7%)	3,791 (4.6%)	171 (3.8%)
*Patient Socioeconomic Status (SES)* *^a^*						
PDI = 1	28,924 (17.2%)	4,707 (18.5%)	14,672 (17.9%)	3,725 (19.5%)	13,428 (16.9%)	666 (15.4%)
PDI = 2	35,201 (20.9%)	6,001 (23.6%)	17,876 (21.8%)	4,759 (24.9%)	16,434 (20.7%)	959 (22.2%)
PDI = 3	33,840 (20.1%)	5,214 (20.5%)	16,286 (19.9%)	4,076 (21.4%)	16,813 (21.2%)	886 (20.5%)
PDI = 4	32,075 (19.0%)	4,617 (18.1%)	15,681 (19.1%)	3,527 (18.5%)	15,648 (19.7%)	907 (21.0%)
PDI = 5	32,293 (19.2%)	4,073 (16.0%)	14,621 (17.8%)	3,006 (15.7%)	17,048 (41.5%)	908 (21.0%)
*Visit Year*						
2004–2010	62,434 (37.0%)	7,645 (30.0%)	28,035 (34.2%)	5,796 (29.4%)	33,419 (40.4%)	1,630 (36.1%)
2011–2018	106,186 (63.0%)	17,816 (70.0%)	53,912 (65.8%)	13,934 (70.6%)	49,283 (59.6%)	2,880 (63.9%)
**Factors Related to Need**						
*History of Concussion-Related EOC*						
Single EOC	123,244 (73.1%)	15,298 (60.1%))	57,198 (69.8%)	11,739 (59.5%)	63,796 (77.1%)	2,953 (65.5%)
>1 EOC	45,376 (26.9%)	10,163 (39.9%)	24,749 (30.2%)	7,991 (40.5%)	18,906 (22.9%)	1,557 (34.5%)
*Location of Index Visit*						
ED	n/a	n/a	50,998 (62.2%)	10,467 (53.0%)	82,702 (100.0%)	4,510 (100.0%)
PO	n/a	n/a	30,949 (37.8%)	9,263 (47.0%)	n/a	n/a
*Diagnosis*						
Concussion	81,947 (48.6%)	19,730 (77.5%)	81,947 (100.0%)	19,730 (100.0%)	n/a	n/a
Other specified injuries of head	8,178 (4.9%)	369 (1.5%)	n/a	n/a	8,178 (9.9%)	369 (8.2%)
Postconcussional syndrome	3,971 (2.4%)	1,221 (4.8%)	n/a	n/a	n/a	n/a
Unspecified injury of head	74,524 (44.2%)	4,141 (16.3%)	n/a	n/a	74,524 (90.1%)	4,141 (91.8%)

^a^ Does not sum to 100.0% due to the exclusion of small dissemination areas in the calculation of the PDI.

#### 3.1.1. Factors associated with follow-up care

All predisposing, enabling, and need-based factors identified *a priori* were significantly associated with follow-up care in univariate models and included in the multivariable model. Crude odds ratios (OR), adjusted OR (aOR), and 95% confidence intervals (CI) are reported in Table 6. Analyses demonstrated low multicollinearity among explanatory variables (mean cohort VIF 1.08 and 1.03 for concussion). ROC curves indicated satisfactory discrimination with Area Under the Curve (AUC) of 0.73 for the cohort and 0.64 for the concussion sub-analysis.

**Table 6:** Univariate and multivariable logistic regression analyses to measure the association between predisposing, enabling, and need factors and follow-up care for pediatric concussion, post-concussion syndrome, other specified injuries of head, and unspecified injury of head diagnoses in Alberta between April 1, 2004 and March 31, 2018.

Explanatory Variable(s)	Total Cohort (*N* = 194,081)				Concussion Diagnosis (*n* = 101,677)			
	Univariate Analysis		Multivariable Analysis		*Univariate Analysis*		*Multivariable Analysis*	
	Crude OR (95% CI)	*P*-Value	Adjusted OR (95% CI)	*P*-Value	Crude OR (95% CI)	*P*-Value	Adjusted OR (95% CI)	*P*-Value
**Predisposing Factors**								
*Sex*								
Female	Reference Category	Reference Category	Reference Category	Reference Category
Male	1.11 (1.08–1.15)	<0.001	1.04 (1.01–1.07)	0.019	1.06 (1.02–1.09)	0.001	1.05 (1.02–1.09)	0.003
*Age at Index Visit (Years)*								
Age (Years, 0–17)	1.14 (1.13–1.14)	<0.001	1.10 (1.09–1.10)	<0.001	1.09 (1.08–1.09)	<0.001	1.09 (1.08–1.09)	<0.001
**Enabling Factors**								
*Community Type (Patient Residence)*								
Metropolitan	Reference Category	Reference Category	Reference Category	Reference Category
Metro-Influenced Areas	1.44 (1.39–1.49)	<0.001	1.31 (1.26–1.36)	<0.001	1.34 (1.28–1.39)	<0.001	1.33 (1.28–1.39)	<0.001
Urban	0.78 (0.74–0.82)	<0.001	0.87 (0.82–0.92)	<0.001	0.87 (0.82–0.92)	<0.001	0.91 (0.85–0.97)	0.004
Urban-Influenced Areas	1.11 (1.02–1.20)	0.013	1.11 (1.01–1.21)	0.023	1.06 (0.96–1.16)	0.264	1.12 (1.02–1.23)	0.024
Rural	1.00 (0.96–1.03)	0.89	1.02 (0.98–1.07)	0.245	0.97 (0.93–1.02)	0.235	1.10 (1.04–1.15)	<0.001
Large Rural Centres and Surrounding Areas	0.96 (0.90–1.02)	0.18	1.03 (0.96–1.10)	0.462	1.00 (0.92–1.08)	0.906	1.08 (0.99–1.17)	0.073
Remote	0.65 (0.59–0.71)	<0.001	0.89 (0.81–0.99)	0.026	0.84 (0.74–0.94)	0.003	0.97 (0.85–1.10)	0.607
*Area-Based Socioeconomic Status (Patient SES)*								
PDI quintile (1–5)	0.93 (0.92–0.94)	<0.001	0.97 (0.96–0.98)	<0.001	1.06 (1.05–1.07)	<0.001	0.96 (0.95–0.97)	<0.001
**Need-Based Factors**								
*History of Concussion-Related EOC*								
Single EOC	Reference Category	Reference Category	Reference Category	Reference Category
>1 EOC	1.80 (1.75–1.86)	<0.001	1.45 (1.41–1.49)	<0.001	1.57 (1.52–1.62)	<0.001	1.43 (1.38–1.48)	<0.001
*Location of Index Visit*								
ED	n/a	n/a	Reference Category	Reference Category
PO	n/a	n/a	1.46 (1.41–1.50)	<0.001	1.49 (1.44–1.54)	<0.001
*Visit year*								
2004–2010	Reference Category	Reference Category	Reference Category	Reference Category
2011–2018	1.37 (1.33–1.41)	<0.001	1.18 (1.15–1.22)	<0.001	1.25 (1.21–1.29)	<0.001	1.15 (1.11–1.19)	<0.001
*Diagnosis*								
Concussion	Reference Category	Reference Category	n/a	n/a
Other specified injuries of head	0.19 (0.17–0.21)	<0.001	0.30 (0.27–0.34)	<0.001	n/a	n/a
Postconcussional syndrome	1.28 (1.20–1.36)	<0.001	1.04 (0.97–1.11)	0.312	n/a	n/a
Unspecified injury of head	0.23 (0.22–0.24)	<0.001	0.36 (0.35–0.37)	<0.001	n/a	n/a

#### 3.1.2. Total cohort analysis

Males (aOR = 1.04, 95% CI: 1.01–1.07) and older children and adolescents (aOR = 1.10, 95% CI: 1.09–1.10 per additional year of age) were more likely to receive follow-up care (Table 6). Community residence was also significantly associated with follow-up care; compared with children living in metropolitan areas, those living in metro-influenced or urban-influenced areas were more likely to receive follow-up care (aOR = 1.31, 95% CI: 1.26–1.36 and aOR = 1.11, 95% CI: 1.01–1.21, respectively), while children living in urban or remote communities were less likely to receive follow-up care (aOR = 0.87, 95% CI: 0.82–0.92 and aOR = 0.89, 95% CI: 0.81–0.99, respectively) (Table 6). Area-based SES was also associated with follow-up care. As SES decreased, the likelihood of follow-up care also decreased (aOR = 0.97, 95% CI: 0.96–0.98) (Table 6). Patients with more than one EOC during the study period were more likely to receive follow-up care (aOR = 1.45, 95% CI: 1.41–1.49) as were EOC that occurred between 2011 and 2018 (aOR = 1.18, 95% CI: 1.15–1.22) (Table 6). Finally, diagnosis was associated with follow-up care, such that EOC that began with a diagnosis other than concussion were significantly less likely to receive follow-up care (aOR = 0.30, 95% CI: 0.27–0.34 for “other specified” and aOR = 0.36, 95% CI: 0.35–0.37 for “unspecified” injuries) (Table 6).

#### 3.1.3. Concussion EOC sub-analysis

Among individuals diagnosed with concussion, males (aOR = 1.05, 95% CI: 1.02–1.09) and older children and adolescents (aOR = 1.09, 95% CI: 1.08–1.09 per year) were more likely to receive follow-up care (Table 6). Compared with metropolitan areas, patients living in metro-influenced, urban-influenced, or rural areas were more likely to receive follow-up care (aOR = 1.33, 95% CI: 1.28–1.39; aOR = 1.12, 95% CI: 1.02–1.23; and aOR = 1.10, 95% CI: 1.04–1.15, respectively), while patients living in urban communities were less likely (aOR = 0.91, 95% CI: 0.85–0.97) (Table 6). As in the total cohort analysis, area-based SES was also associated with follow-up care, and as SES decreased, the likelihood of follow-up care also decreased (aOR = 0.96, 95% CI: 0.95–0.99) (Table 6). Patients with more than one EOC in the study period were significantly more likely to receive follow-up care (aOR = 1.43, 95% CI: 1.38–1.48), as were patients whose EOC began in PO (aOR = 1.49, 95% CI: 1.44–1.54) (Table 6). As in the total cohort analysis, initial EOC from 2011 to 2018 were significantly more likely to receive follow-up care than those from 2004 to 2010 (aOR = 1.15, 95% CI: 1.11–1.19) (Table 6).

## 4. Discussion

Even though we don't have clinical information to gauge need for follow-up care, clinical practice guidelines for concussion recommend all patients follow-up in outpatient settings 7–10 days after initial injury and diagnosis (22). While follow-up care increased during the study period, it remained relatively rare among children and youth diagnosed with concussion in Alberta. To inform service delivery, this study identified factors associated with follow-up care after pediatric concussion and associated diagnoses. Our results show that there are both system-level and patient-level factors to consider regarding health service utilization after concussion.

### 4.1. Predisposing factors

Males were more likely to receive follow-up care than females, although as our previous report showed, the proportion of females receiving follow-up care increased more dramatically over the study period (1). While males typically engage in more collision sports and more risky behaviours than females, increasing their likelihood of sustaining a concussion, females report more acute and persistent symptoms (23, 24). The increased symptom reporting in females (25, 26) might suggest they would receive follow-up care more often than males, but this is not what our findings demonstrate. The higher rate of follow-up among males may reflect a referral bias, whereby physicians refer males for follow-up more often than females. Alternatively, because males more often take part in collision sports, they may require medical clearance prior to return to play, necessitating more follow-up visits.

Age was another important predisposing factor, with each additional year of age increasing the adjusted odds of follow-up by 10%. Rates of other head injury diagnoses (i.e., not concussion) were higher among younger children, especially those under five years of age (1). This may be partly because young children are less likely to be diagnosed with concussion given the challenges involved (27, 28), but also because clinical practice guidelines for pediatric concussion management are targeted to those over the age of five years (22, 29). On the other hand, the lower rate of follow-up care may reflect evidence that younger children are less likely to demonstrate persistent symptoms (30). Age-related differences in the mechanism of concussion also may have an influence: Among older children sports and recreation-related activities are the primary setting for concussions (31). Current guidelines recommend that all athletes with a concussion obtain medical clearance prior to returning-to-sport (6, 32–34). If older youth are more likely to sustain a concussion during sport and require medical clearance to return to play, the likely result is more follow-up visits. In contrast, given that most concussions sustained by children under age five are the result of falls (35), the likelihood of follow-up care may be lower.

### 4.2. Enabling factors

While most children still were not receiving follow-up care at the end of the study period, the likelihood of follow up increased after 2011. Consistent with other studies (36, 37), the increase may reflect growing awareness of concussion resulting from heightened media attention (9, 35). However, as shown in our previous report, this increase was not consistent across all community types or levels of SES (1). As follow up care was less common remote areas, this may be associated with access, whether it be enhanced access in certain areas or impeded access in others. Access to health care can be limited in remote and particularly rural areas, often as a result of scarcity of health care providers, in those regions (38).

Patients from areas of lower SES also were less likely to receive follow up care, regardless of community type. Although Canada's health care system ensures access to care regardless of income, other geographic, socioeconomic, and cultural barriers still exist that prevent people from receiving or obtaining care (39–45). Additional study to uncover and explore barriers to follow-up care after pediatric concussion is needed to ensure that attention and resources are best deployed. While low SES has been associated with a higher prevalence of risky behaviours that could result in a concussion (46, 47), high SES has been associated with increased involvement in organized sports for children and youth (48). Over the study period, the proportion of EOC with follow-up increased the most among patients from lower SES (1). This could reflect increased access to care in these areas, a relative greater increase in concussion awareness, or growth in the number of children of lower SES participating in organized sports. Thus, further study into the etiology of pediatric concussion and follow-up with consideration of injury mechanism is warranted.

### 4.3. Need-based factors

Factors related to need include both perceived and evaluated (or objective and subjective) needs related to health service use (13, 15). History of concussion-related EOC can impact perceived need due to a patient's knowledge of or experience with the health care system or previous concussions. We found in a previous study that patients with a previous EOC had twice the rate of increase in follow up care over the study period relative to those with a first EOC (1) which likely signals growing awareness of the increased risk of persistent post-concussive symptoms among children with multiple concussions, as well as the potential need for multidisciplinary care in such cases (1, 34, 48, 49).

Evaluated need in this context includes diagnosis and location of health service use, assuming that, on some level, more severe cases are receiving initial care in the ED (we lack an injury severity variable or scale to validate or score severity). EOC that began with a non-concussion head injury diagnosis were less likely to involve follow-up care. This is not surprising given clinical practice guidelines relate specifically to concussion diagnoses. However, the finding does emphasize the importance of accurate diagnosis, as children and adolescents diagnosed with other head injuries will likely not receive the same management that they would if diagnosed with concussion, or their parents may not perceive a need for follow-up care. Clearer diagnostic standards are needed for concussion in young children, to increase the likelihood of appropriate follow-up care.

Children with a concussion diagnosis whose first visit occurred in outpatient (PO) settings were more likely to have a follow up visit than those whose first visit was in the ED, which may reflect greater continuity of care with primary care physicians. While considerable gaps in knowledge and inconsistent application of clinical practice guidelines have been documented among both ED and primary-care physicians (7, 8), differences in the types of patients that initially receive care in the ED vs. PO may also help explain differences in follow-up care. For example, we found previously that the proportion of patients receiving care in the ED increased as SES decreased, and the proportion of patients receiving concussion-related care in the ED was highest in rural (and particularly remote) areas (1). Thus, the increased likelihood of follow-up in EOCs that began in PO may be partly related to the availability of care in outpatient or community health service for rural and remote communities and those of lower SES.

### 4.4. Limitations

This study has several limitations. First, our findings may not generalize to jurisdictions beyond Alberta, Canada due to local factors that may be related to access to health care. In addition, the nature of administrative health data is such that its quality and validity are unknown (50). Because we did not perform a validation in this study, miscoding, non-specific diagnoses, and overrepresentation of certain diagnoses are possible. In addition, data entry issues could have hampered the linkage process. However, studies indicate that the quality of administrative data in Canada is high (50–52) and our robust case definition using a range of head injury diagnoses helped to capture potential misdiagnoses. Also, the possibility of misclassification arises from our definitions of cases or EOC; however, previous population-based studies have used similar approaches (10, 18) and our EOC definition was developed in conjunction with study clinicians. In addition, our data only pertained to services covered under the publicly-funded provincial health care plan, meaning our results do not account for care provided in non-ED or PO settings, outside the public system, or by allied health practitioners. The average proportion of health expenditure over the 2004–2018 period in Alberta was 72.0% public and 28.0% private (insurance or out-of-pocket) (53).

Because administrative health databases do not contain socioeconomic information, the PDI was used to characterize area-based SES. The PDI is an area-based measure of material deprivation; in the absence of individual-level data, area-level aggregate data are considered a valid proxy (54) and the measure is the most widely used and cited area-based SES indicator in Canada (55). Patient postal codes were probabilistically linked to census data to determine the PDI. In many rural areas, postal codes cover larger geographical areas than urban postal codes (56) and the assumed homogeneity in SES could be incorrect. Because our study includes all records across the province, our sample size is likely large enough that these impacts are not significant. In addition, some small rural areas have missing data, likely due to small populations. This has implications for the interpretation of SES in these communities, as the PDI may not accurately reflect the true proportion of patients. However, only 3.6% of all records (*n* = 8,322) were missing a PDI quintile and so the impact is thought to be minimal. Finally, administrative health data provides limited or no information regarding mechanism or severity of injury and no data regarding clinical outcomes. Thus, future studies should explore the associations between SES, mechanism of injury, and follow-up care.

## 5. Conclusion

Despite these limitations, our linkage of databases capturing all inpatient and outpatient services, robust case and EOC definitions, and 14 years of population-based data provides a basis for a better understanding of follow-up care among children and adolescents post-concussion. While follow-up care is not as routine as advised in clinical practice guidelines, it has increased over time. The rate of follow-up care is associated with patient age and sex and history of concussion-related EOC, where a patient lives (community type and area-based SES), and when and where the index visit occurred. Follow-up care is less frequent in non-metropolitan areas and in areas of lower SES, suggesting a need to increase access. Health care practitioners need guidance about how and when to use the concussion diagnosis with younger children to ensure they receive appropriate follow-up care post injury. A better understanding of which children are more likely to receive follow-up care, as well as how and when they do so, is an important step in aligning practice with follow-up guidelines.

## Data Availability

The original contributions presented in the study are included in the article/Supplementary Materials, further inquiries can be directed to the corresponding author.

## References

[1] Wittevrongel K Barrett O Couloigner I Bertazzon S Hagel B Schneider KJ Longitudinal trends in incidence and health care use for pediatric concussion in Alberta. Canada Peditric Res. (2022). 10.1038/s41390-022-02214-5 PMC10172117 36085365

[2] Tator CH. Let’s standardize the definition of concussion and get reliable incidence data. Can J Neurol Sci. (2009) 36:405–6. 10.1017/S031716710000771X 19650349

[3] Marsh A Marsh D Marsh JP. Management of concussion in the pediatric patient. J Pediatr Heal Care. (2013) 27:499–504. 10.1016/j.pedhc.2012.12.014 23522559

[4] Kinnaman K Mannix R Comstock R Meehan W. Management of pediatric patients with concussion by emergency medicine physicians. Pediatr Emerg Care. (2014) 30:458–61. 10.1097/PEC.0000000000000161 24977993

[5] Tarimala A Singichetti B Yi H Huang L Doerschuk R Tiso M Initial emergency department visit and follow-up care for concussions among children with medicaid. J Pediatr. (2019) 206:178–83. 10.1016/j.jpeds.2018.10.021 30442410

[6] Ellis MJ Bauman S Cowle S Fuselli P Tator CH. Primary care management of concussion in Canada. Paediatr Child Heal. (2019) 24:137–42. 10.1093/pch/pxy171 PMC6519658 31110450

[7] Zemek R Eady K Moreau K Farion KJ Solomon B Weiser M Canadian pediatric emergency physician knowledge of concussion diagnosis and initial management. Can J Emerg Med. (2015) 17:115–22. 10.1017/cem.2014.38 25927255

[8] Zemek R Eady K Moreau K Farion KJ Solomon B Weiser M Knowledge of paediatric concussion among front-line primary care providers. Pediatr Child Heal. (2014) 19:475–80. 10.1093/pch/19.9.475 PMC4235448 25414583

[9] Zemek RL Grool AM Rodriguez Duque D DeMatteo C Rothman L Benchimol EI Annual and seasonal trends in ambulatory visits for pediatric concussion in Ontario between 2003 and 2013. J Pediatr. (2017) 181:222–228.e2. 10.1016/j.jpeds.2016.10.067 27843008

[10] Fridman L Scolnik M Macpherson A Rothman L Guttmann A Grool AM Annual trends in follow-up visits for pediatric concussion in emergency departments and Physicians’ offices. J Pediatr. (2018) 192:184–8. 10.1016/j.jpeds.2017.09.018 29150146

[11] Haran HP Bressan S Oakley E Davis GA Anderson V Babl FE. On-field management and return-to-play in sports-related concussion in children: are children managed appropriately? J Sci Med Sport. (2016) 19:194–9. 10.1016/j.jsams.2015.02.009 25772997

[12] Mercuri M Sherbino J Sedran RJ Frank JR Gafni A Norman G. When guidelines don’t guide: the effect of patient context on management decisions based on clinical practice guidelines. Acad Med. (2015) 90:191–6. 10.1097/ACM.0000000000000542 25354075

[13] Andersen R Newman JF. Societal and individual determinants of medical care utilization in the United States. Milbank Meml Fund Q Heal Soc. (1973) 51:95–124. 10.2307/3349613 4198894

[14] Andersen RM. Revisiting the behavioral model and access to medical care: does it matter? J Health Soc Behav. (1995) 36:1–10. 10.2307/2137284 7738325

[15] Aday LA Andersen R. A framework for the study of access to medical care. Health Serv Res. (1974) 9:208–20. 10.1080/08912963.2016.1278444 4436074 PMC1071804

[16] Benchimol EI Smeeth L Guttmann A Harron K Moher D Peteresen I The REporting of studies Conducted using Observational Routinely-collected health Data (RECORD) statement. PLoS Med. (2015) 12:1–22. 10.1371/journal.pmed.1001885 PMC4595218 26440803

[17] Centers for Disease Control and Prevention. Report to congress on mild traumatic brain injury in the United States: steps to prevent a serious public health problem (2003). 10.3181/00379727-102-25369

[18] Macpherson A Fridman L Ba MS Corallo A Mdcm AG Macpherson A A population-based study of paediatric emergency department and office visits for concussions from 2003 to 2010. Pediatr Child Heal. (2014) 19:543–6. 10.1093/pch/19.10.543 PMC4276389 25587234

[19] Alberta Health Services & Alberta Health. Official standard geographic areas (2018). https://open.alberta.ca/dataset/a14b50c9-94b2-4024-8ee5-c13fb70abb4a/resource/70fd0f2c-5a7c-45a3-bdaa-e1b4f4c5d9a4/download/official-standard-geographic-area-document.pdf

[20] Alberta Health Services. Pampalon deprivation index: user guide for Alberta (2016). p. 1–27.

[21] StataCorp. Stata statistical software: release 15 (2017).

[22] Alberta Health Services. Provincial clinical knowledge topic: concussion, pediatric - emergency version 1 (2020). https://extranet.ahsnet.ca/teams/policydocuments/1/klink/et-klink-ckv-frailty-seniors-acute-care.pdf

[23] Bertz K Divine J Foss K Heyl R Ford K Myer G. Sex-specific differences in the severity of symptoms and recovery rate following sports-related concussion in young athletes. Phys Sportsmed. (2013) 41:58–63. 10.3810/psm.2013.05.2015 23703518

[24] Koerte IK Schultz V Sydnor VJ Howell DR Guenette JP Dennis E Sex-related differences in the effects of sports-related concussion: a review. J Neuroimaging. (2020) 30:387–409. 10.1111/jon.12726 32533752 PMC8221087

[25] Merritt V Arnett P. Premorbid predictors of postconcussion symptoms in collegiate athletes. J Clin Exp Neuropsychol. (2014) 36:1098–111. 10.1080/13803395.2014.983463 25493542

[26] Preiss-Farzanegan S Chapman B Wong T Wu J Bazarian J. The relationship between gender and postconcussion symptoms after sport-related mild traumatic brain injury. PM&R. (2009) 1:245–53. 10.1016/j.pmrj.2009.01.011 19627902 PMC5237580

[27] Boutis K Weerdenburg K Koo E Schneeweiss S Zemek R. The diagnosis of concussion in a pediatric emergency department. J Pediatr. (2015) 166:1214–1220.e1. 10.1016/j.jpeds.2015.02.013 25919731

[28] Boutis K Gravel J Freedman SB Craig W Tang K DeMatteo CA The diagnosis of concussion in pediatric emergency departments: a prospective multicenter study. J Emerg Med. (2018) 54:757–65. 10.1016/j.jemermed.2018.02.041 29685472

[29] Ontario Neurotrauma Foundation. Guidelines for diagnosing and managing pediatric concussion (2014). 10.1136/archdischild-2014-307252 26297033

[30] Karlin AM. Concussion in the pediatric and adolescent population: “different population, different concerns”. PM&R. (2011) 3:S369–79. 10.1016/j.pmrj.2011.07.015 22035679

[31] Haarbauer-Krupa J Arbogast KB Metzger KB Greenspan AI Kessler R Curry AE Variations in mechanisms of injury for children with concussion. J Pediatr. (2018) 197:241–248.e1. 10.1016/j.jpeds.2018.01.075 29627189 PMC6029621

[32] Ontario Neurotrauma Foundation. Guideline for concussion/mild traumatic brain injury & persistent symptoms 3rd ed. (2018). p. 1–13. https://braininjuryguidelines.org/concussion/fileadmin/media/adult-concussion-guidelines-3rd-edition.pdf

[33] Parachute. Canadian guideline on concussion in sport (2017).

[34] McCrory P Meeuwisse W Dvořák J Aubry M Bailes J Broglio S Consensus statement on concussion in sport—the 5th international conference on concussion in sport held in Berlin, October 2016. Br J Sports Med. (2017) 51:838–47. 10.1136/bjsports-2017-097699 28446457

[35] Stewart TC Gilliland J Fraser DD. An epidemiologic profile of pediatric concussions: identifying urban and rural differences. J Trauma Acute Care Surg. (2014) 76:736–42. 10.1097/TA.0b013e3182aafdf5 24553542

[36] Rose SC Weber KD Collen JB Heyer GL. The diagnosis and management of concussion in children and adolescents. Pediatr Neurol. (2015) 53:108–18. 10.1016/j.pediatrneurol.2015.04.003 26088839

[37] Hardesty W Singichetti B Yi H Leonard JC Yang J. Characteristics and costs of pediatric emergency department visits for sports- and recreation-related concussions, 2006–2014. J Emerg Med. (2019) 56:571–9. 10.1016/j.jemermed.2019.01.001 30857833

[38] Barrett O. Measuring accessibility to primary health care across the urban-rural continuum in the Province of Alberta (2016). 10.1017/CBO9781107415324.004

[39] Arora S Kurji AK Tennant MTS. Dismantling sociocultural barriers to eye care with tele-ophthalmology: lessons from an Alberta cree community. Clin Investig Med. (2013) 36:57–64. 10.25011/cim.v36i2.19567 23544606

[40] Huot S Ho H Ko A Lam S Tactay P MacLachlan J Identifying barriers to healthcare delivery and access in the circumpolar north: important insights for health professionals. Int J Circumpolar Health. (2019) 78:1–8. 10.1080/22423982.2019.1571385 30696379 PMC6352934

[41] Jong M Mendez I Jong R. Enhancing access to care in northern rural communities via telehealth. Int J Circumpolar Health. (2019) 78:1–3. 10.1080/22423982.2018.1554174 31066652 PMC6508052

[42] King M Smith A Gracey M. Indigenous health part 2: the underlying causes of the health gap. Lancet. (2009) 374:76–85. 10.1016/S0140-6736(09)60827-8 19577696

[43] Oosterveer TM Young TK. Primary health care accessibility challenges in remote indigenous communities in Canada’s North. Int J Circumpolar Health. (2015) 74:1–7. 10.3402/ijch.v74.29576 PMC4623283 26507717

[44] Ellis MJ Russell K. The potential of telemedicine to improve pediatric concussion care in rural and remote communities in Canada. Front Neurol. (2019) 10:1–12. 10.3389/fneur.2019.00840 PMC6688625 31428043

[45] Gelberg L Andersen RM Leake BD. The behavioral model for vulnerable populations: application to medical care use and outcomes for homeless people. Health Serv Res. (2000) 34:1273–302. PMID: ; PMCID: 10654830 PMC1089079

[46] Whitman S Coonley-Hoganson R Desai BT. Comparative head trauma experiences in two socioeconomically different Chicago-area communities: a population study. Am J Epidemiol. (1984) 119:570–80. 10.1093/oxfordjournals.aje.a113774 6711546

[47] Kraus JF Fife D Ramstein K Conroy C Cox P. The relationship of family income to the incidence, external causes, and outcomes of serious brain injury, San Diego county, California. Am J Public Health. (1986) 76:1345–7. 10.2105/AJPH.76.11.1345 3766837 PMC1646731

[48] White P McTeer W. Socioeconomic status and sport participation at different developmental stages during childhood and youth: multivariate analyses using Canadian national survey data. Sociol Sport. (2012) 29:186–209. 10.1123/ssj.29.2.186

[49] Meehan W Bachur R. Sport-related concussion. Pediatrics. (2009) 123:114–23. 10.1542/peds.2008-0309 19117869

[50] Mazzali C Paganoni AM Ieva F Marsella C Maistrello M Agostoni O Methodological issues on the use of administrative data in healthcare research: the case of heart failure hospitalizations in Lombardy region, 2000–2012. BMC Health Serv Res. (2016) 16:234. 10.1186/s12913-016-1489-0 27391599 PMC4939041

[51] Lucyk K Tang K Quan H. Barriers to data quality resulting from the process of coding health information to administrative data: a qualitative study. BMC Health Serv Res. (2017) 17:1–10. 10.1186/s12913-017-2697-y 29166905 PMC5700659

[52] Canadian Institute for Health Information. CIHI data quality study of the 2009–2010 discharge abstract database (2012). p. 1–115. Available at: https://secure.cihi.ca/free_products/Reabstraction_june19revised_09_10_en.pdf (Revised 2012).

[53] Canadian Institute for Health Information. National health expenditure trends. *Ser B Total Heal Expend by source Financ by Prov Canada Table B11, Table B21, Table B31* (2022). Available at: https://www.cihi.ca/en/national-health-expenditure-trends.

[54] Diez-Roux A. Bringing context back into epidemiology: variables and fallacies in multilevel analysis. Am J Public Health. (1998) 88:216–22. 10.2105/AJPH.88.2.216 9491010 PMC1508189

[55] Pampalon R Hamel D Gamache P Raymond G. A deprivation index for healthy planning. Chronic Dis Can. (2009) 29:178–91. 10.24095/hpcdp.29.4.05 19804682

[56] Pichora E Polsky JY Catley C Perumal N Jin J Allin S. Comparing individual and area-based income measures: impact on analysis of inequality in smoking, obesity, and diabetes rates in Canadians 2003–2013. Can J Public Heal. (2018) 109:410–8. 10.17269/s41997-018-0062-5 PMC6964493 29981091

